# Resting-State EEG Power and Aperiodic Activity in Individuals with Mild Cognitive Impairment and Cognitively Healthy Controls

**DOI:** 10.3390/brainsci15121305

**Published:** 2025-12-03

**Authors:** Teresa S. Warren, Shraddha A. Shende, Jaya Ashrafi, Grace M. Clements, Raksha A. Mudar

**Affiliations:** 1 Neuroscience Program, University of Illinois Urbana-Champaign, Champaign, IL 61801, USA; teresaw3@illinois.edu; 2 Communication Sciences and Disorders, Illinois State University, Normal, IL 61790-4720, USA; sashend@ilstu.edu; 3 Brain and Cognitive Science, University of Illinois Urbana-Champaign, Champaign, IL 61820, USA; jayaashrafi@outlook.com; 4 Air Force Research Laboratory, Wright-Patterson Air Force Base, Dayton, OH 45433, USA; gmclements30@gmail.com; 5 Speech and Hearing Science, University of Illinois Urbana-Champaign, Champaign, IL 61820, USA

**Keywords:** mild cognitive impairment, resting-state EEG, aperiodic activity, 1/*f*, cognition, older adults

## Abstract

Background: Resting-state electroencephalography (EEG) abnormalities have been widely studied in mild cognitive impairment (MCI) and are linked to cognition. Traditionally, research has focused on the absolute power spectrum, which includes both aperiodic (1/*f*) and periodic components. However, fewer studies have examined aperiodic (1/*f*) and periodic components separately and their relationship to cognition in cognitively healthy older adults and individuals with MCI. Objectives: This study examined (i) group differences in resting-state absolute power, 1/*f*-adjusted power, and 1/*f* slope in individuals with MCI and cognitively healthy controls, and (ii) associations between cognition and 1/*f*-adjusted power and slope within each group. Methods: Nineteen individuals were included in each group. All participants completed eyes-open resting-state EEG and a cognitive battery assessing global functioning, cognitive control, verbal fluency, naming, and episodic memory. Absolute power and 1/*f*-adjusted power in theta (4–7 Hz), alpha (8–12 Hz), and beta (13–30 Hz) bands and 1/*f* slope were extracted. Results: No group differences emerged in the resting-state measures. In the controls, a flatter 1/*f* slope was linked to worse verbal fluency, but no significant associations were observed in the MCI group. Conclusions: Although there were no group differences, the link between 1/*f* slope and cognition in the controls highlights the value of separately examining periodic and aperiodic brain activity to better understand cognition in individuals with MCI and healthy aging.

## 1. Introduction

Mild cognitive impairment (MCI) is defined by cognitive deficits that are disproportionate for an individual’s age and educational background, yet not severe enough for a dementia diagnosis [1,2,3]. Approximately 23.7% of older adults live with MCI globally [4], with this number expected to rise with the global population aging [5]. Individuals with MCI are at an increased risk of developing Alzheimer’s disease (AD) and related dementias [1], underscoring the importance of early screening and diagnosis, particularly given the recent pharmacological advances in slowing MCI progression.

Neural markers hold promise for early detection as neuropathological changes underlying AD begin decades before observable behavioral symptoms emerge [6]. Electroencephalography (EEG) is an inexpensive, non-invasive tool that may aid early detection of MCI. It can be used to examine spontaneous neural oscillations at rest and event-related activity across different frequency bands: delta (0.5–4 Hz), theta (4–7 Hz), alpha (8–12 Hz), beta (13–30 Hz), and gamma (30–100 Hz). Both event-related [7,8,9,10,11] and resting-state EEG [12,13] have shown promise in distinguishing neurophysiological abnormalities in individuals with MCI from cognitively healthy controls. However, resting-state EEG offers the distinct advantage of broad applicability compared to event-related EEG, as resting-state data collection is straightforward and requires minimal participant effort.

Differences in resting-state power have been consistently reported between individuals with AD dementia compared to cognitively healthy older adults [12,14,15,16,17,18,19,20,21]. However, findings in individuals with MCI relative to controls have been less consistent. For instance, some studies have reported differences in absolute theta power [18,22,23,24,25], and to a lesser extent in alpha [22] and beta power [23], in individuals with MCI relative to cognitively healthy controls, whereas others have reported no differences in absolute power across the spectrum [26,27,28]. Thus, it remains unclear whether resting-state EEG can reliably distinguish individuals with MCI from cognitively healthy older adults.

One potential source distinguishing individuals with MCI from cognitively healthy controls is 1/*f* activity, also known as aperiodic/non-oscillatory activity. Resting-state EEG consists of both periodic activity, which represents the oscillatory or rhythmic neuronal firing that contributes to narrowband peaks, and 1/*f* activity, which involves irregular, asynchronous neuronal firing that contributes to a broadband effect where power is inversely proportional to frequency [29,30,31]. Although 1/*f* activity was previously dismissed as functionally irrelevant “neural noise,” it is now thought to provide valuable insights into the efficiency of cognitive functioning [32]. This perspective differs from traditional approaches used in resting-state EEG studies, which often aggregate periodic and aperiodic activity.

Recent research has begun to delineate age-dependent variations in 1/*f* activity across the adult lifespan [33]. Studies have found that older adults have a flatter, or noisier, 1/*f* power spectrum compared to young adults [30,34,35,36,37]. Although studies comparing individuals with MCI to controls have generally found no differences in 1/*f* slope and 1/*f*-adjusted narrowband power [38,39,40], the limited number of studies underscores a critical gap and highlights the need for further studies in this area.

The potential of resting-state EEG as a non-invasive biomarker of cognitive health is further supported by emerging evidence that suggests that it can predict cognitive performance [41]. For example, resting-state delta, theta, and alpha power have been linked to performance on measures of cognitive control and perceptual speed [42,43], verbal memory, verbal recall, and attention [43,44,45], as well as speed of processing [34] in cognitively healthy older adults. In individuals with MCI, resting-state delta and alpha power have been linked to performance on the Mini-Mental Status Examination (MMSE), a measure of global cognition [15,46,47], while beta power has been associated with delayed memory recall [48]. Links between 1/*f* activity, and its impact on narrowband activity, and cognition have also been reported. Specifically, 1/*f* slope has been found to mediate age-related deficits in visual working memory [30], while 1/*f*-adjusted theta and alpha power have been linked to different aspects of cognitive control [35] in typical cognitive aging. However, whether these associations extend to atypical cognitive aging, particularly to MCI, remains unknown.

This study aimed to address the gaps in the current understanding of the periodic and aperiodic activity difference in individuals with MCI relative to controls by investigating group differences in resting-state absolute power, 1/*f*-adjusted power, and 1/*f* slope in a quasi-exploratory manner. Additionally, correlations between 1/*f*-adjusted power and 1/*f* slope and cognitive performance were explored in each of the groups through both hypothesis-driven and exploratory analyses. Based on the existing literature [18,22,23,24,25], we expected the MCI group to have higher power in the theta band and lower power in alpha and beta bands relative to cognitively healthy older adults. Given the literature on cognitive functioning and theta [49,50,51,52], alpha [53,54,55,56], and beta [48,57,58,59] bands, we hypothesized that 1/*f*-adjusted theta power would correlate with measures of cognitive control, 1/*f*-adjusted alpha power would correlate with measures of naming and verbal fluency, and 1/*f*-adjusted beta power would correlate with measures of delayed recall on episodic memory (see Section 2. Materials and Methods for details).

## 2. Materials and Methods

### 2.1. Participants

Participants included 19 individuals with MCI and 19 cognitively healthy older adult controls who were comparable in age and level of education (see Table 1 for demographics). Both groups included native English speakers with normal or corrected vision and hearing. Participants were excluded if they had a history of neurological disorders, major psychiatric illnesses or substance abuse, depressive symptoms (scored ≤ 5 on Geriatric Depression Scale [60] or scored ≤ 10 on Beck Depression Inventory-II [61]), or history of major health conditions.

Participants in the MCI group met the following criteria [62]: (i) subjective memory difficulties corroborated by a reliable informant, (ii) episodic memory impairment on the delayed recall subtest from Wechsler Memory Scale-III logical memory [63], (iii) relatively preserved independence, (iv) level of cognitive and functional impairment insufficient to meet dementia diagnosis, and (v) a score of 0.5 on the Clinical Dementia Rating Scale [64]. This sample included participants with multi-domain, amnestic MCI. Two participants were on a stabilized dose of cholinesterase inhibitors for a minimum of three months at the time of participation in this study. All MCI participants involved in this study provided written informed consent in accordance with protocols approved by the Institutional Review Boards of The University of Texas at Dallas and in accordance with the Helsinki Declaration of 1975, as revised in 1983.

Participants in the cognitively healthy control group had no cognitive complaints, all obtaining a score of ≥26 on the Montreal Cognitive Assessment (MoCA) [65], and performed on par with their age and education on cognitive measures (see Table 1). Written informed consent was obtained from all control participants involved in this study in accordance with protocols approved by the Institutional Review Boards of the University of Illinois Urbana-Champaign and the Carle Institutional Review Board, and in accordance with the Helsinki Declaration of 1975, as revised in 1983.

### 2.2. EEG Acquisition and Processing

Participants underwent EEG recordings in an eyes-open resting-state condition. During the recordings, participants were seated in a comfortable chair with a computer screen positioned in front of them. They were instructed to stare directly at a black fixation cross (a “+” presented in the center of the display). Continuous EEG recordings were gathered for a minimum of 5 min using a 64-electrode Neuroscan QuikCap (Compumedics Neuroscan, Charlotte, NC, USA) in combination with a Neuroscan SynAmp2/SynAmpRT amplifier (Compumedics Neuroscan, Charlotte, NC, USA) and Scan v4.5 software. Data were sampled at 1 kHz with an online bandpass filter of DC-200 Hz, and electrode impedances were typically maintained below 10 kΩ throughout the recording. The reference electrode was positioned at the midline between electrodes Cz and CPz. A vertical electrooculogram (VEOG) was collected from electrodes placed above and below the left eye to account for ocular artifacts.

Offline processing of raw EEG data was performed in Neuroscan Edit v4.5 software. A high-pass filter with a 0.15 Hz cutoff was applied to the data, and eye blink artifacts were corrected via spatial filter transform, which uses a regression-based approach that identifies and removes blink components while retaining signals of interest [66]. Poorly functioning electrodes were identified through visual inspection and those determined to be noisy were removed from further analysis (MCI: *M* = 0.58 noisy electrodes per participant, *SD* = 0.77; controls: *M* = 1.68 noisy electrodes per participant, *SD* = 1.20). Cleaned data with removed blinks were imported into MATLAB 2021a for further preprocessing. First, the removed electrodes were interpolated by computing the average of all electrodes based on spherical splines fitted to the data. Then, EEG data were re-referenced to the average potential over the entire scalp. Lastly, the continuous EEG data were divided into epochs of 1000 ms. Any epochs contaminated by physiological artifacts (e.g., muscular, head movements, etc.) were visually identified and removed from subsequent analyses. The average number of accepted epochs was 248.00 epochs (*SD* = 44.53) in the MCI group and 217.42 epochs (*SD* = 48.18) in the control group. Student’s *t*-test revealed a trending group difference in the number of accepted epochs: *t*(36) = −1.98, *p* = 0.056.

**Figure 1:**
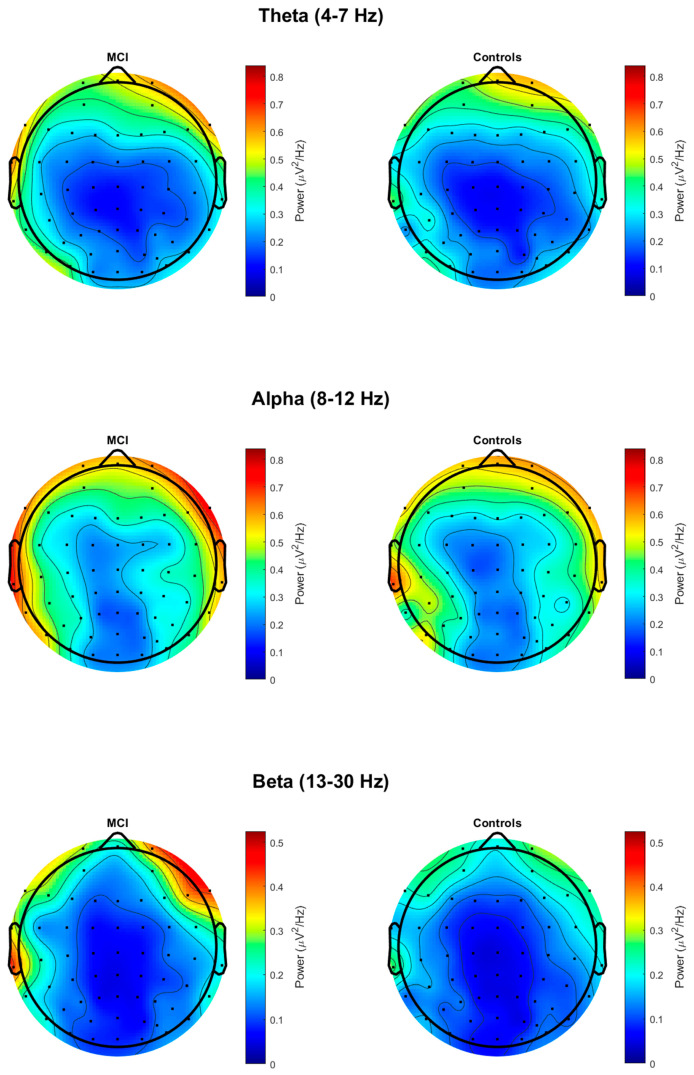
Topographic maps of absolute power at theta, alpha, and beta in MCI and control groups.

Power spectral densities were calculated using EEGLAB [67] and FieldTrip [68] in MATLAB 2021a. A fast Fourier transformation (FFT) was performed using FieldTrip’s ft_freqanalysis() with the following parameters: cfg.method = ‘mtmfft,’ and cfg.taper = ‘hanning’. The FFT was applied to each 1000 ms epoch at each electrode and across a frequency range of 1–30 Hz with a frequency resolution of 1 Hz. The single-trial FFTs were then averaged over the epochs, resulting in one FFT per electrode per participant. Scalp distribution maps of absolute power for theta, alpha, and beta are presented in Figure 1.

Spectra were parameterized to determine the aperiodic parameters, 1/*f* slope and intercept, to control for the confounding effects of non-oscillatory activity on oscillatory power estimates. Using an approach previously described [35,69], aperiodic activity was modeled using a theory-driven, censored regression approach. To model the aperiodic 1/*f* component of the power spectrum, we transformed the raw spectral data from linear space to log–log space and applied simple linear regression using Matlab’s fitlm() function. Prior to fitting the data, we removed power values from 4 to 13 Hz by setting them to NaNs. This removal was carried out to omit narrowband oscillatory activity in the theta and alpha range, which is known to produce prominent peaks in the spectrum that would impact estimation of aperiodic activity [70].

Subsequently, the 1/*f* slope and intercept were estimated and retained for analysis. We selected 1/*f* slope as the primary aperiodic dependent variable given that the prior literature on aperiodic components and aging has shown reliable relationships with this parameter [30,36,37]. Next, the estimated aperiodic fit was subtracted from the absolute power spectrum. This resulted in a ‘flattened’ power spectrum uncontaminated with aperiodic activity, which will henceforth be referred to as 1/*f*-adjusted power. For both absolute power and 1/*f*-adjusted power, we chose to examine the average power at the fronto-central electrodes (F1, Fz, F2, FC1, FCz, FC2, C1, Cz, and C2) for the theta band (4–7 Hz) and parietal electrodes (CP1, CPz, CP2, P1, Pz, and P2) for the alpha (8–12 Hz) and beta (13–30 Hz) bands. We also calculated individual alpha frequency (IAF) to detect whether subtle changes occurred in alpha between the MCI and control groups. Global power was calculated across all electrodes, and then peak power across an extended alpha range from 7 to 14 Hz was identified for each participant. No significant difference in IAF (*p* = 1.000) was observed between the MCI (*M* = 10.11, *SD* = 1.89) and control (*M* = 10.11, *SD* = 1.41) groups. As a result, we used the fixed range from 8 to 12 Hz for all participants. Our analysis focused on these specific electrode clusters given that theta oscillations are strongest in fronto-central regions [71] while alpha and beta oscillations are most prominent in parietal regions [19,72]. To maintain consistency with our absolute power analyses, we explored group differences in 1/*f* slope in the same fronto-central and parietal regions as well.

### 2.3. Cognitive Measures

Participants completed a battery of cognitive measures (see Table 1 for group performance). The MMSE [73] was used in the MCI group and the MoCA [65] was used in the control group to measure global cognitive function. In the MCI group, MMSE scores were converted into equivalent MoCA scores based on established conversions [74]. Both groups completed the following cognitive measures: Wechsler Memory Scale-III logical memory immediate and delayed recall subtests [63] to assess episodic memory; Trail Making Test-A (TMT-A) and Trail Making Test-B (TMT-B) [75] to assess cognitive control; Boston Naming Test (BNT) [76] to assess naming; and Controlled Oral Word Association Test (COWAT) letter fluency and category fluency subtests [77] to assess verbal fluency and cognitive control. Five participants from the control group did not complete the Wechsler III logical memory subtests. In addition, two participants from the MCI group did not complete the BNT. Missing scores on cognitive measures were imputed using their respective groups’ mean scores.

### 2.4. Statistical Analyses

Data were analyzed using R 4.4.1 (R Core Team, 2024). Student’s *t*-test was conducted using the t.test() function to examine group differences in demographics and performance on cognitive measures. A Chi-square test using the chisq.test() function was used to examine group differences in sex. Group differences in resting-state EEG absolute power, 1/*f*-adjusted power, and 1/*f* slope at each frequency band of interest at its respective electrode cluster (theta at fronto-central electrodes, alpha at parietal electrodes, and beta at parietal electrodes) were examined using a one-way Analysis of Variance (ANOVA) with aov(). To account for the unbalanced sex ratio between groups (see Table 1), sex was added as a covariate in the ANOVA models. Pearson’s correlations using the cor.test() function were conducted to examine the relationship between cognitive measures with resting-state 1/*f*-adjusted power and 1/*f* slope within each group separately. Specifically, we examined whether (i) 1/*f*-adjusted theta power correlated with performance on TMT-B and COWAT for letter fluency, (ii) 1/*f*-adjusted alpha power correlated with performance on BNT and COWAT for category fluency, and (iii) 1/*f*-adjusted beta power correlated with performance on MMSE for the MCI group and MoCA for the control group, as well as the logical memory delayed subtest. For correlations between 1/*f* slope and cognitive measures, we examined whether (i) 1/*f* slope at the fronto-central electrodes correlated with performance on TMT-B and COWAT for letter fluency and (ii) 1/*f* slope at parietal electrodes correlated with performance on BNT, COWAT for category fluency, MMSE/MoCA, and the logical memory delayed subtest. Post hoc partial correlation analyses were conducted using the pcor.test() function to control for sex. These analyses examined the relationship between 1/*f*-adjusted power, 1/*f* slope, and performance on cognitive measures controlling for sex as a covariate. Alpha was fixed at 0.05 for all analyses.

## 3. Results

### 3.1. Demographics and Cognitive Measures

Table 1 displays demographic information and performance on cognitive measures for each group. Groups did not differ by age or education. However, groups did differ in sex, with more females in the control group relative to the MCI group. As expected, groups differed on the MoCA, Wechsler Memory Scale-III logical memory subtests (both immediate and delayed), BNT, and COWAT for letter fluency, in which the MCI group performed worse compared to the control group. The groups did not differ on any other cognitive measures.

### 3.2. Resting-State EEG Group Differences

The group means and *p*-values for the resting-state measures of absolute power, 1/*f*-adjusted power, and 1/*f* slope are reported in Table 2. There were no significant differences between groups in absolute power or 1/*f*-adjusted power in theta at the fronto-central electrodes, alpha at the parietal electrodes, or beta at the parietal electrodes (*p* > 0.05). There were no significant group differences in the 1/*f* slope at the fronto-central or parietal electrodes (*p* > 0.05). Given the significant group difference in sex, post hoc analyses were conducted to include sex as a covariate (see Supplementary Table S1). However, these analyses did not change the results.

### 3.3. Associations Between Resting-State EEG Measures and Cognitive Measures

#### 3.3.1. Correlations with 1/*f*-Adjusted Power and Cognitive Measures

In the MCI group, there were no significant correlations between resting-state 1/*f*-adjusted power and cognitive measures (see Table 3). In the controls, we observed a significant negative correlation between 1/*f*-adjusted theta power at the fronto-central electrodes and COWAT for letter fluency, *r*(17) = −0.48, *p* = 0.036 (see Figure S1). We also observed a significant positive correlation between 1/*f*-adjusted beta power at the parietal electrodes and MoCA in the controls, *r*(17) = 0.46, *p* = 0.048 (see Figure S2), as well as a trending positive correlation between 1/*f*-adjusted beta power at the parietal electrodes and logical memory delayed scores, *r*(17) = 0.45, *p* = 0.051.

In post hoc analyses, partial correlations were conducted to control for sex in the previously observed associations within the control group. After adjusting for sex, the association between 1/*f*-adjusted theta power at the fronto-central electrodes and COWAT letter fluency was no longer significant, *r*(16) = −0.26, *p* = 0.294. Similarly, the positive correlation between 1/*f*-adjusted beta power at the parietal electrodes and MoCA was attenuated and did not reach significance, although it remained as a trend, *r*(16) = 0.46, *p* = 0.053.

#### 3.3.2. Correlations with 1/*f* Slope and Cognitive Measures

In a set of exploratory analyses, we investigated the relationship between resting-state 1/*f* slope and cognitive measures. In the MCI group, there were no significant correlations between resting-state 1/*f* slope and cognitive measures (*p* > 0.05; see Table 4). In the controls, a significant negative correlation was found between 1/*f* slope at the fronto-central electrodes and COWAT for letter fluency, *r*(17) = −0.63, *p* = 0.004 (see Figure S3). There were no other significant correlations between 1/*f* slope and cognitive measures in the controls.

In a post hoc analysis, a partial correlation was conducted to control for sex in the previously observed significant correlation within the control group. After adjusting for sex, the negative correlation between 1/*f* slope at the fronto-central electrodes and COWAT letter fluency remained significant, *r*(17) = −0.47, *p* = 0.045.

## 4. Discussion

This study examined resting-state EEG differences in both periodic (1/*f*-adjusted power) and aperiodic (1/*f* slope) components, as well as absolute power, in individuals with MCI and cognitively healthy controls. We also explored how cognitive performance relates to periodic and aperiodic components of resting-state EEG within each group. The findings revealed no group differences in resting-state absolute power, 1/*f*-adjusted power, or 1/*f* slope. However, in the controls, there were associations between 1/*f* slope and performance on letter fluency. Notably, these associations were not present in the MCI group. These findings underscore the importance of separately examining periodic (1/*f*-adjusted power) and aperiodic (1/*f* slope) components of neural activity to better understand the relationship between resting-state EEG and cognitive health in older adults.

There were no differences between groups in resting-state absolute power at the theta, alpha, or beta bands, aligning with findings of some previous studies [26,27,28]. However, others have reported group differences, particularly in theta [18,22,24,25] and to a lesser extent in alpha [22] and beta [18] bands, between individuals with MCI and controls (for review, see Babiloni et al. (2021) [12]). Methodological differences may explain some of these discrepancies. It is well documented that brain state dynamics differ in eyes-closed and eyes-open resting conditions [78,79]. The studies that observed group differences used eyes-closed resting-state EEG data [22,23,24,25], whereas the current study used an eyes-open condition. For example, within our sample, alpha power at the parietal electrodes was markedly reduced, a pattern not uncommon in eyes-open resting-state EEG [15,78,79,80], but may have been further influenced by our use of a centroparietal midline reference relative to studies with linked mastoids as a reference. Our findings suggest that eyes-open resting-state EEG data alone may have limited utility in distinguishing individuals with MCI from cognitively healthy older adults. However, recent findings suggest that examining changes in resting-state EEG following a cognitive task, relative to the pre-task baseline, may offer a more sensitive approach for differentiating MCI from cognitively healthy controls [81,82,83]. Applying such an approach to eyes-open resting-state EEG data could potentially enhance its utility and should be explored in future work.

Also, abnormalities in resting-state EEG become more pronounced in later stages of cognitive decline and therefore may vary across the spectrum of MCI. In the current study, participants from the MCI group were almost evenly split between “early” (*n* = 10) and “late” (*n* = 9) MCI stages based on their logical memory delayed subtest scores. Unfortunately, the majority of studies typically only report MMSE scores, without providing results from other cognitive measures [18,22,25], with the exception of Tomasello et al. (2023) [24], which limits comparison of our findings in relation to other studies. In relation to other studies reporting MMSE scores of 25–27 for MCI groups [18,22,24,25], our sample included individuals with milder impairment, as evidenced by an average MMSE score of 28. It remains unclear whether our findings differ from previous studies because our MCI participants had relatively higher global cognitive functioning or due to the heterogeneity introduced by combining early and late MCI within a relatively small sample of 19 individuals. This may have contributed to variability and limited statistical power. Future studies with larger, well-characterized samples controlled for cognitive abilities are necessary to verify these null findings.

No significant differences emerged between the MCI and control groups, even when components of the periodic activity (1/*f*-adjusted power) and aperiodic activity (1/*f* slope) were separately examined, which is similar to the findings from a handful of other studies [38,39,40]. These results remained consistent even after accounting for sex-related group differences. Our findings add to the limited body of evidence suggesting that aperiodic components may not be substantially altered in MCI. However, this interpretation should be considered cautiously given the small sample size and sex differences between the groups. Given the paucity of research on 1/*f* dynamics in MCI and dementia, further studies with larger samples are needed to determine whether alterations in 1/*f* emerge only in more advanced stages of neurodegeneration or in specific variants of MCI subtypes.

Lastly, in examining the relationship between performance on cognitive measures and periodic (1/*f*-adjusted power) and aperiodic (1/*f* slope) activity, we found associations within the control group, but not in the MCI group. In particular, higher resting-state 1/*f*-adjusted theta power and flatter 1/*f* slopes at the fronto-central electrodes were linked to poorer performance on COWAT for letter fluency, a measure of verbal fluency and cognitive control, in the controls. This aligns with prior work that has linked frontal theta oscillations with cognitive control processes [44,49,51,84,85,86,87,88]. However, the association between periodic theta power and letter fluency was not observed after adjusting for sex, suggesting potential differences in letter fluency processing between cognitively healthy older men and women [89,90]. We also found a marginally significant correlation between 1/*f*-adjusted beta power and MoCA scores, which attenuated to a trend level after controlling for sex. The attenuation of these associations after adjusting for sex suggests that sex differences likely drove the observed link between periodic theta oscillations and letter fluency, as well as periodic beta oscillations and global cognition. In contrast, the relationship between the 1/*f* slopes at the fronto-central electrodes and letter fluency performance remained significant even after adjusting for sex, indicating that aperiodic features may not be as sensitive to sex and could therefore be a useful biomarker of cognitive changes in cognitively healthy older adults. Although not examined here, 1/*f* offset has also been linked to cognitive control [35] and verbal fluency [91] in cognitively healthy young and older adults. Overall, our findings suggest that aperiodic activity is less influenced by sex differences than periodic theta and beta activity.

In contrast to the controls, we did not observe any significant relationship between resting-state EEG activity and cognitive performance in the MCI group. These findings differ from previous studies that have reported relationships between resting-state EEG power and MMSE [15,46,47] and delayed memory recall [48]. However, these studies did not control for aperiodic activity, which may have influenced the observed associations. Taken together, our findings may reflect altered neurophysiological–cognitive coupling in individuals with MCI. Emerging work suggests that the 1/*f* slope reflects the brain’s excitation–inhibition (E/I) balance, with inhibition producing a steeper slope and excitation producing a flattened slope [92]. Our findings indicate that steeper 1/*f* slopes are related to preserved cognitive functioning. Along the MCI-to-AD continuum, there is a shift in excitation, which corresponds with a lower aperiodic exponent [93,94,95]. One might speculate that the lack of significant relationships between cognitive measures and resting-state periodic and aperiodic activity in the MCI group could reflect cortical hyperexcitability. The apparent decoupling between neural dynamics and cognition may therefore indicate disrupted network integrity, where an altered E/I balance weakens the association between 1/*f* and cognitive performance. Further research is needed to confirm whether these patterns represent an early marker of cortical network disruption in individuals with MCI.

This study has several limitations. The relatively small sample size and uneven sex distribution across groups may have reduced statistical power to detect meaningful group differences. Additionally, the MCI group was heterogenous and characterized by a higher education and relatively mild cognitive deficits compared to other studies, which limits the generalizability of these findings. Furthermore, we assessed only eyes-open resting-state EEG data and did not conduct whole-brain analyses given the exploratory nature of this study, which may restrict direct comparisons with studies that use eyes-closed data with whole-brain analyses.

## 5. Conclusions

The current study did not find group differences in resting-state EEG between individuals with MCI and cognitively healthy older adult controls, even after separating periodic activity (1/*f*-adjusted power) and the aperiodic slope, and adjusting for an unbalanced sex ratio. However, we did observe associations between aperiodic activity and cognitive performance in the controls but not in the MCI group. These results emphasize the value of disentangling periodic and aperiodic components of brain activity to gain deeper insights into cognitive functioning in older adults.

## Figures and Tables

**Table 1:** Demographics and performance on cognitive measures.

	MCI	Controls	*p*-Value
Demographics			
Total N	19	19	--
Age (yrs)	68.84 (7.28)	69.58 (6.75)	0.748
Education (yrs)	17.21 (1.51)	16.58 (2.57)	0.363
Sex	8 F/11 M	16 F/3 M	0.019 *
Cognitive Measures			
MMSE	28.05 (1.47)	--	--
MoCA	23.53 (2.99)	28.11 (1.29)	<0.001 *
LM: Immediate	10.53 (1.84)	17.14 (1.69)	<0.001 *
LM: Delayed	8.63 (2.19)	16.14 (1.91)	<0.001 *
TMT-A (S)s.)	29.74 (9.25)	26.32 (7.14)	0.211
TMT-B (S)s.)	76.37 (27.24)	65.79 (14.60)	0.147
BNT	27.24 (2.37)	28.47(1.12)	0.050 *
COWAT: Letter Fluency	41.47 (8.10)	52.00 (7.96)	<0.001 *
COWAT: Category Fluency	20.16 (6.82)	22.32 (5.15)	0.279

Each cell represents group mean (standard deviation), except for sex. *p*-values were derived from Student’s *t*-tests for all variables, except for sex, which was derived from Chi-square test. *: significant *p*-values (*p* < 0.05). MMSE: Mini-Mental State Exam. MoCA: Montreal Cognitive Assessment. LM: Logical memory. TMT: Trail Making Test. BNT: Boston Naming Test. COWAT: Controlled Oral Word Association Test. In TMT, the symbol S represents time expressed in seconds.

**Table 2:** Group differences in resting-state absolute power, 1/*f*-adjusted power, and 1/*f* slope.

	MCI	Controls	Main Effect of Group
Absolute Power			
Theta (fronto-central)	0.2106 (0.2032)	0.1882 (0.1770)	*F*(1, 36) = 0.12; *p* = 0.726
Alpha (parietal)	0.2136 (0.2641)	0.2142 (0.2797)	*F*(1, 36) = 0.00; *p* = 0.995
Beta (parietal)	0.0601 (0.0513)	0.0554 (0.0550)	*F*(1, 36) = 0.07; *p* = 0.793
1/*f*-Adjusted Power			
Theta (fronto-central)	−0.0347 (0.0476)	−0.0552 (0.0844)	*F*(1, 36) = 0.81; *p* = 0.373
Alpha (parietal)	0.0135 (0.0533)	−0.0056 (0.0629)	*F*(1, 36) = 0.97; *p* = 0.332
Beta (parietal)	0.0003 (0.0009)	−0.0002 (0.0020)	*F*(1, 36) = 0.89; *p* = 0.353
1/*f* Slope			
Fronto-central	−0.0464 (0.0405)	−0.0621 (0.0611)	*F*(1, 36) = 0.83; *p* = 0.368
Parietal	−0.0384 (0.0408)	−0.0564 (0.0663)	*F*(1, 36) = 0.96; *p* = 0.333

Each cell represents group mean (standard deviation). Absolute power was quantified in µV^2^/Hz and 1/f-adjusted power computed as log_10_(µV^2^/Hz).

**Table 3:** Correlations between resting-state 1/*f*-adjusted power and cognitive measures.

	MCI	Controls
1/*f*-Adjusted Power: Theta (Fronto-Central)		
TMT-B	r(17) = −0.22, *p* = 0.361	r(17) = −0.17, *p* = 0.478
COWAT: Letter Fluency	r(17) = 0.04, *p* = 0.864	r(17) = −0.48, *p* = 0.036 *
1/*f*-Adjusted Power: Alpha (Parietal)		
BNT	r(17) = 0.06, *p* = 0.818	r(17) = 0.11, *p* = 0.665
COWAT: Category Fluency	r(17) = −0.17, *p* = 0.495	r(17) = −0.11, *p* = 0.641
1/*f*-Adjusted Power: Beta (Parietal)		
MMSE/MoCA ^1^	*r*(17) = −0.09, *p* = 0.709	*r*(17) = 0.46, *p* = 0.048 *
LM: Delayed	*r*(17) = 0.12, *p* = 0.620	*r*(17) = 0.45, *p* = 0.051

^1^ MMSE was used for the MCI group and MoCA was used for controls. *: significant *p*-values (*p* < 0.05). TMT: Trail Making Test. COWAT: Controlled Oral Word Association Test. BNT: Boston Naming Test. MMSE: Mini-Mental State Examination. MoCA: Montreal Cognitive Assessment. LM: Logical memory.

**Table 4:** Correlations between 1/*f* slope and cognitive measures.

	MCI	Controls
1/*f* Slope: Fronto-Central		
TMT-B	r(17) = −0.08, *p* = 0.750	*r*(17) = −0.02, *p* = 0.920
COWAT: Letter Fluency	r(17) = 0.11, *p* = 0.650	*r*(17) = −0.63, *p* = 0.004 *
1/*f* Slope: Parietal		
BNT	*r*(17) = 0.22, *p* = 0.375	*r*(17) = −0.29, *p* = 0.236
COWAT: Category Fluency	*r*(17) = −0.10, *p* = 0.687	*r*(17) = −0.31, *p* = 0.189
MMSE/MoCA ^1^	*r*(17) = 0.17, *p* = 0.481	*r*(17) = 0.21, *p* = 0.385
LM: Delayed	*r*(17) = 0.20, *p* = 0.402	*r*(17) = 0.16, *p* = 0.509

^1^ MMSE was used for the MCI group and MoCA was used for controls. *: significant *p*-values (*p* < 0.05). TMT: Trail Making Test. COWAT: Controlled Oral Word Association Test. BNT: Boston Naming Test. MMSE: Mini-Mental State Examination. MoCA: Montreal Cognitive Assessment. LM: Logical memory.

## Data Availability

The data presented in this study are available upon request to the corresponding author. The data are not publicly available due to privacy reasons.

## Supplementary Materials

The following supporting information can be downloaded at https://www.mdpi.com/article/10.3390/brainsci15121305/s1, Figure S1: The association between 1/*f*-adjusted power at theta (fronto-central electrodes) and letter fluency in controls; Figure S2: The association between 1/*f*-adjusted power at beta (parietal electrodes) and Montreal Cognitive Assessment in controls; Figure S3: The association between 1/*f* slope at fronto-central electrodes and letter fluency in controls. Table S1. Sex-adjusted group differences in resting-state absolute power, 1/*f*-adjusted power, and 1/*f* slope.

## Abbreviations

The following abbreviations are used in this manuscript:

MCI	Mild cognitive impairment
AD	Alzheimer’s disease
EEG	Electroencephalography
MMSE	Mini-Mental Status Examination
MoCA	Montreal Cognitive Assessment
TMT-A	Trail Making Test-A
TMT-B	Trail Making Test-B
BNT	Boston Naming Test
COWAT	Controlled Oral Word Association Test
LM	Logical memory
