# Supplementary material for: Resting-State EEG Power and Aperiodic Activity in Individuals with Mild Cognitive Impairment and Cognitively Healthy Controls
Source: Brain Sci. 2025 Dec 3;15(12):1305. doi: 10.3390/brainsci15121305 (PMC12730939; doi:10.3390/brainsci15121305)

Supplementary Materials

Table S1. Sex-adjusted group differences in resting-state absolute power, 1/*f*-adjusted power, and 1/*f* slope.

	Main Effect of Group	Main Effect of Sex
Absolute Power		
Theta (fronto-central)	$F(1, 35) = 0.13$; $p = 0.724$	$F(1, 35) = 1.59$; $p = 0.216$
Alpha (parietal)	$F(1, 35) = 0.00$; $p = 0.994$	$F(1, 35) = 2.73$; $p = 0.108$
Beta (parietal)	$F(1, 35) = 0.08$; $p = 0.786$	$F(1, 35) = 3.51$; $p = 0.069$
1/*f*-Adjusted Power		
Theta (fronto-central)	$F(1, 35) = 0.84$; $p = 0.365$	$F(1, 35) = 2.26$; $p = 0.142$
Alpha (parietal)	$F(1, 35) = 0.95$; $p = 0.336$	$F(1, 35) = 0.43$; $p = 0.514$
Beta (parietal)	$F(1, 35) = 0.86$; $p = 0.359$	$F(1, 35) = 0.11$; $p = 0.738$
1/*f* Slope		
Fronto-central	$F(1, 35) = 0.86$; $p = 0.359$	$F(1, 35) = 2.48$; $p = 0.124$
Parietal	$F(1, 35) = 0.97$; $p = 0.330$	$F(1, 35) = 1.45$; $p = 0.236$

Each cell represents group mean (standard deviation). Absolute power was quantified in $\mu V^2/Hz$ and 1/f-adjusted power computed as $\log_{10}(\mu V^2/Hz)$.

Figure S1. 1/*f*-Adjusted Power at Theta (Fronto-Central Electrodes) and Letter Fluency in Controls.

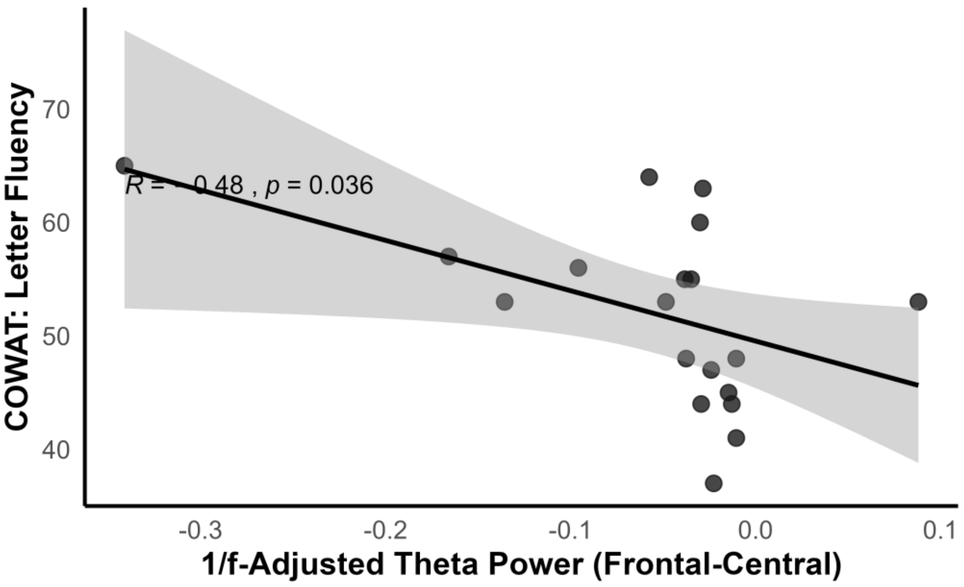

Figure S2. 1/*f*-Adjusted Power at Beta (Parietal Electrodes) and Montreal Cognitive Assessment in Controls.

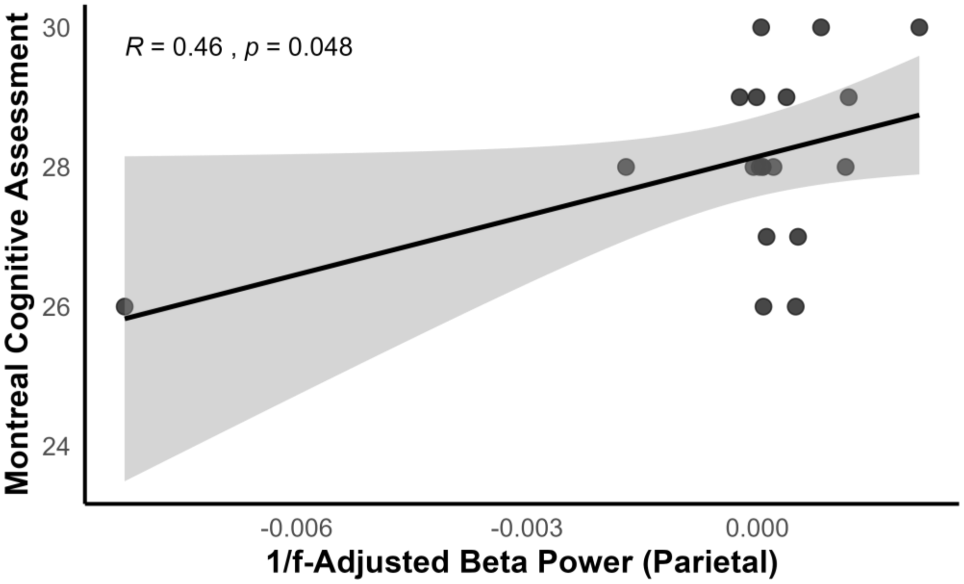

Figure S3. 1/*f* Slope at Fronto-Central Electrodes and Letter Fluency in Controls.

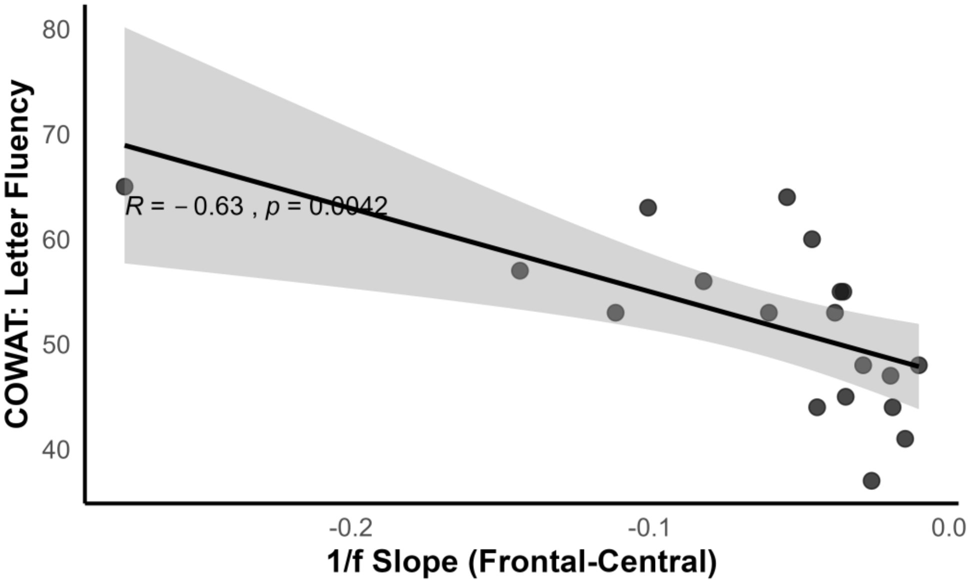

Supplement: Supplementary file 1 [file brainsci-15-01305-s001.zip › brainsci-3984064-supplementary.pdf]
